# Chlorogenic Acid Improves Late Diabetes through Adiponectin Receptor Signaling Pathways in *db/db* Mice

**DOI:** 10.1371/journal.pone.0120842

**Published:** 2015-04-07

**Authors:** Shasha Jin, Cuiqing Chang, Lantao Zhang, Yang Liu, Xianren Huang, Zhimin Chen

**Affiliations:** Institute of Sports Medicine, Peking University Third Hospital, Beijing, China; University of Catanzaro Magna Graecia, ITALY

## Abstract

The aim of this study was to examine the effects of chlorogenic acid (CGA) on glucose and lipid metabolism in late diabetic *db/db* mice, as well as on adiponectin receptors and their signaling molecules, to provide evidence for CGA in the prevention of type 2 diabetes. We randomly divided 16 female *db/db* mice into *db/db*-CGA and *db/db*-control (CON) groups equally; db/m mice were used as control mice. The mice in both the *db/db*-CGA and db/m-CGA groups were administered 80 mg/kg/d CGA by lavage for 12 weeks, whereas the mice in both CON groups were given equal volumes of phosphate-buffered saline (PBS) by lavage. At the end of the intervention, we assessed body fat and the parameters of glucose and lipid metabolism in the plasma, liver and skeletal muscle tissues as well as the levels of aldose reductase (AR) and transforming growth factor-β1 (TGF-β1) in the kidneys and measured adiponectin receptors and the protein expression of their signaling molecules in liver and muscle tissues. After 12 weeks of intervention, compared with the *db/db*-CON group, the percentage of body fat, fasting plasma glucose (FPG) and glycosylated hemoglobin (HbA1c) in the *db/db*-CGA group were all significantly decreased; TGF-β1 protein expression and AR activity in the kidney were both decreased; and the adiponectin level in visceral adipose was increased. The protein expression of adiponectin receptors (ADPNRs), the phosphorylation of AMP-activated protein kinase (AMPK) in the liver and muscle, and the mRNA and protein levels of peroxisome proliferator-activated receptor alpha (PPAR-α) in the liver were all significantly greater. CGA could lower the levels of fasting plasma glucose and HbA1c during late diabetes and improve kidney fibrosis to some extent through the modulation of adiponectin receptor signaling pathways in *db/db* mice.

## Introduction

Diabetes has become a worldwide epidemic. In 2011, there were 366 million people with diabetes, and this number is expected to rise to 552 million by 2030 [1]. In China, the age-standardized prevalence of total diabetes and prediabetes was 9.7%, amounting to 92.4 million Chinese adults with diabetes, and this disease has already become a major public health problem [2]. Type 2 diabetes and its complications are a serious hazard to human health. The main causes of premature death are cardiovascular disease, blindness, kidney disease, neuropathy and amputation. As the occurrence and development of type 2 diabetes are closely related to unhealthy lifestyles (such as unreasonable dietary structure and limited physical activity), finding natural bio-active ingredients in frequently consumed foods to attenuate diabetes has become urgent and significant.

CGA is a type of phenolic acid created by the condensation of caffeic acid and quinic acid, also known as 5-coffee quinic acid (5-CQA, the IUPAC numbering). It is widely available in seeds, fruits, vegetables and coffee drinks and is also the main functional ingredient in herbal honeysuckle and eucommia. Systematic reviews and meta-analyses of prospective cohort studies indicate that habitual coffee consumption is associated with a substantially lower risk (RR 0.70~0.40) of type 2 diabetes; this effect is dose-responsive [3–6]. A crowd randomized controlled intervention experiment showed that either the oral administration of CGA or drinking coffee rich in CGA can improve glucose tolerance and decrease plasma glucose in both healthy and overweight people [7–9]. In addition, the long-term consumption of coffee rich in CGA can help facilitate weight loss in obese people [10]. The plant extract containing CGA helps improve glucose and lipid metabolism, lowers plasma glucose and C-reactive protein levels, and improves liver function in diabetic patients [11, 12]. Animal experiments suggest that CGA has beneficial effects on glucose and lipid metabolism disorders in high fat diet-induced mice and rats and can also improve type 2 diabetes in genetically predisposed animals [13–17]. Recent studies claim that CGA modulates glucose-6-phosphatase (G-6-P) activity to reduce hepatic glucose output [18, 19]. CGA can increase glucose uptake by skeletal muscle cells by activating AMPK [20], and it can also decrease the triglyceride content in plasma, liver and muscle and enhance insulin sensitivity by promoting the expression of PPAR-α in the liver [17]. However, the mechanism underlying CGA-mediated improvements in insulin sensitivity requires further research. Adiponectin is generally accepted as an insulin sensitizer. Moreover, adiponectin and its associated receptors play an important role in improving obesity-related diabetes [21]. Thus, we hypothesize that CGA acts through ADPNR-related signaling pathways to improve glucose and lipid metabolism. In this study we used the *db/db* diabetic mouse model to observe the impact of CGA on the adiponectin receptor and its associated signaling molecules as well as its influence on glucose and lipid metabolism during late diabetes and diabetic nephropathy. Therefore, our study may provide scientific support for using CGA to prevent type 2 diabetes.

## Materials and Methods

### Ethics statement

Animals were treated humanely, using approved procedures in accordance with the guidelines of the Peking University Health Science Center Animal Care and Use Committee. The study was approved by the Animal Care and Use Committee at the Peking University Health Science Center.

### Animals

Five to six-week-old 20–25 g female C57BL/BKS *db/db* mice (n = 16) were divided into *db/db*-control (CON) (n = 8) and *db/db*-CGA groups (n = 8). As a control, five to six-week-old female db/m mice were also randomly divided into either a db/m-CON group (n = 8) or a db/m-CGA group (n = 8). Animals in the CGA groups were treated once daily with CGA (CGA:5-CQA, 99% purity, extracted from green coffee seeds, Acros Ltd., USA) (80 mg/kg/d). Van Dam R M. et al. showed that habitual coffee consumption was inversely related to type 2 diabetes and that CGA intake was approximately 1000 mg/day in habitual coffee drinkers, equivalent to 14 mg/kg body weight daily for a man of 70 kg body weight. The dose for db mice is generally 5–7 times that for humans, approximately 70–100 mg/kg body weight daily. In this study, the dose of 80 mg/kg body weight daily was lavaged for 12 weeks, and the control groups were treated with PBS in an identical manner. The intervention period was 12 weeks. All mice were kept at constant room temperature (23 ± 2°C) and humidity (55 ± 5%) under a 12 h light/dark cycle. At the end of the experiment, blood samples were taken by enucleating the eyeballs after fasting for 12 h. We then separated the plasma and measured FPG, HbA1c and free fatty acids (FFA) on the same day. Next, the mice were sacrificed with ether anesthesia. Next, the liver, kidney, quadriceps, and visceral adipose tissue surrounding the kidney and ovary were removed, rinsed with physiological saline solution prior to blotting with filter paper, immediately placed into liquid nitrogen and quickly transferred to a -80°C low temperature freezer until use.

### Food intake, body weight and content of visceral fat

During the experiment, food intake was measured daily and body weight (BW) was measured weekly. After 12 weeks of intervention, we measured body length (BL) and final body weight (FBW) prior to sacrifice. After death, we removed and weighed the visceral adipose tissue (VA) surrounding the kidney and ovary. We calculated the percentage of visceral fat (VF %) and Lee's index according to the following formula. VF% = (perirenal + ovarian surrounding) VA / FBW× 100%, Lee's index =$\sqrt[{3}]{BW(g)\times 10^{3}/BL(cm)}$.

### Oral glucose tolerance test

At the end of 8 weeks, all mice were fasted for 12 h before the test. They were given 2.5 g/kg BW glucose in solution by lavage. Then, blood samples were collected from the tail vein at 15, 30, 60 and 120 minutes after glucose loading for measurement using a blood glucose meter (ACCU—CHEK Advantage, Roche, DE). The area under the curve (AUC) values for glucose were calculated using the trapezoidal method.

### Detection of blood biochemistry

Fasting glucose (FPG), total cholesterol (TC), triglycerides (TG), muscle glycogen and liver glycogen were detected by enzymatic colorimetric assays (Biosino Bio-Technology and Science, CHN). Glycated hemoglobin (HbA1c) levels were measured using a commercial kit (Nanjing Jiancheng Bioengineering Institute, CHN). We used the improved copper reagent colorimetric method to determine free fatty acids (FFA) (Nanjing Jiancheng Bioengineering Institute, CHN). Insulin detection was achieved by a competitive assay method with an enzyme-linked immunosorbent assay (ELISA) kit (Rapidbio, USA).

### Detection of adipocytokines and tissue TG

The adiponectin and visfatin contents in VA were determined by ELISA kit (Rapidbio, USA). After chloroform homogenate treatment of liver and muscle tissues, we used an enzymatic colorimetric method to detect liver and muscle TG.

### Detection of aldose reductase (AR) activity in the kidney

Using Vander’s colorimetric method [22] with slight modifications, we added 80–100 mg from part of the kidney tissue to precooled 0.9% saline 1:5 (W/V), homogenized the mixture in a low temperature centrifuge (12000 rpm/min) for 2 h, and pipetted the supernatant fluid for subsequent measurements. The entire process was performed at 4°C. The reaction system totaled 1 ml, with 67 mmol/L of Na+-K+ phosphate buffer (pH = 6.2) 0.4 ml, 10 mmol/L DL-glyceraldehydes (Sigma, USA) 0.1 ml, 100 mmol/L ammonium sulfate 0.3 ml, 0.1 mmol/L coenzyme II (NADPH, Sigma, USA) 0.1 ml, and 0.1 ml tissue supernatant. Control cups used double-distilled water instead of DL-glyceraldehyde as the substrate. NADPH was added to start the reaction at 37°C for 4 min, and then the OD values of NADPH were recorded with a UV260 spectrophotometer at 340 nm, with continuous recording of the OD value every 15 seconds for 3 min. The aldose reductase (AR) activity is the OD value decline per unit of time. The unit of AR is defined as 1 μmol of NADPH per mg of protein consumed per minute (μmol/mg/min).

### Detection of mRNA expression of PPAR-α in liver by RT-PCR

Total RNA was extracted from the liver with Trizol reagent (Life Tech, USA). First-strand cDNA was generated from 3 μg of total RNA in a volume of 20 μL using a reverse-transcription kit (TIANGEN BIOTECH, BJ CHN) with Oligo dT as a primer. The PCR was performed in a final volume of 25 μL containing 2 μg of cDNA, 9.5 μL of double-distilled water, 12.5 μL of PCR master mix (TIANGEN BIOTECH, BJ CHN), and 0.5 μL of each of the primers specific for PPAR-α or GADPH. The primer sequences are as follows: (1) PPAR-α sense primer, 5’-ATGTCCGTGGAGACCGTCA-3’; antisense primer, 5’-GGTTCTTAAGGAACTCGCGTG-3’; (2) GADPH sense primer, 5’-AGGCCGGTGCTGAGTATGTC-3’; and antisense primer, 5’-TGCCTGTTCACCACCTTCT -3’. PPAR-α was amplified under the following conditions: initial denaturation at 94°C for 5 min, followed by 35 cycles of denaturation at 94°C for 30 s, annealing at 55°C for 30 s, and extension at 72°C for 40 s in a GeneAmp PCR system 9700 (PE, USA). As an internal control, GADPH was amplified concomitantly using the same amplification conditions. PCR products (5 μL) were electrophoresed on a 2.0% agarose gel. Band intensity was quantified under UV light using the Sygene Bio-ID system (USA). The level of mRNA expression was expressed as the ratio of band intensity of the target gene relative to that of GADPH.

### Detection of ADPNR-1, ADPNR-2, PPAR-α, pAMPK, G-6-P, GLUT-4, TGFβ-1, and GADPH protein expression by western blot

Total protein was extracted from liver, muscle, and kidney tissues using radioactive immunoprecipitation (RIPA) lysates. Approximately 50–80 μg of protein was run on a discontinuous SDS-PAGE gel and transferred to a nitrocellulose membrane. The membranes were blocked with 5% skim milk in TBST containing 0.05% tween for 2 h and were then incubated with the following primary antibodies overnight at 4°C: (1) rabbit anti-ADPNR-1 (1:500), rabbit anti-ADPNR-2 (1:300), and rabbit anti-TGFβ-1 (1:200) (Santa Cruz Biotech, USA); (2) rabbit anti-PPAR-α (1:200), rabbit anti-G-6-P (1:500), and mouse anti-GLUT-4 (1:500) (Abcam, USA); (3) rabbit anti-AMPKα (1:1500), rabbit anti-Phospho-AMPKα (1:1000) (Cell Signaling Technology, USA); and (4) rabbit anti-GADPH (1:2000, Bioworld Tech). The membrane was washed and incubated at room temperature for 2 h in the dark with goat anti-rabbit fluorescent secondary antibodies (Li-COR Bioscience, USA). The NC membranes were scanned with an Odyssey infrared fluorescence scanner (Li-COR Bioscience, USA). The optical density (OD) of the signals was quantified and expressed as the ratio of OD of the tested proteins (PPAR-α, G-6-P, GLUT-4, ADPNR-1, ADPNR-2, and TGFβ-1) to that of GADPH. The protein expression of AMPK phosphorylation parameters was expressed as the pAMPK and AMPK striped gray value ratio.

### Statistical analysis

Statistical analysis was performed with SPSS 16.0. All data were expressed as the mean±SD. A Student’s t-test for independent-samples was used to compare differences between the two groups. P<0.05 was considered statistically significant.

## Results

### The general condition of *db/db* and db/m mice

During the entire experiment, the food intake of *db/db* mice was significantly higher than that of db/m mice (P <0.05). Compared with db/m mice, BW, Lee's index, VA percentage and plasma FPG, TG, and TC were significantly higher, as well as the whole blood HbA1c and TG contents in the liver and skeletal muscle (P <0.05). Those parameters were in accordance with other spontaneously obese diabetic models (Table 1). However, food intake was not significantly different between the CGA and control groups (P >0.05) in either *db/db* or db/m mice, indicating that CGA has no effect on food intake (Table 1).

**Table 1:** Effect of chlorogenic acid supplementation on physical characteristics and other parameters in plasma, whole blood, liver and muscle in *db/db* and *db/m* mice.*

****Items****		****db/m-CON****	****db/m-CGA****	*****db/db*****-CON****	*****db/db*****-CGA****
Physical parameters	Initial body weight (g)	16.9±0.87^a^	17.0±0.73^a^	26.6±1.05^b^	26.7±0.90^b^
	Final body weight (g)	23.1±0.95^a^	22.3±1.16^a^	58.5±1.25^b^	58.6±1.64^b^
	Lee’s index	14.2±0.22^a^	13.9±0.23^b^	19.1±0.19^c^	19.0±0.23^c^
	Visceral adipose (%)	4.56±0.60^a^	2.66±0.76^b^	12.7±0.34^c^	11.5±1.02^d^
Plasma	FPG (mmol/L)	7.02±0.89^a^	5.52±2.10^a^	20.5±3.96^b^	14.6±4.04^c^
	FFA (mmol/L)	0.88±0.09^a^	0.63±0.18^b^	1.42±0.36^c^	1.35±0.17^c^
	TG (mmol/L)	1.44±0.26^a^	1.29±0.20^a^	1.53±0.14^a^	1.43±0.23^a^
	TC (mmol/L)	2.76±0.28^a^	2.76±0.26^a^	5.21±0.35^b^	4.83±0.45^b^
	Insulin (mIU/L)	9.18±1.02^a^	20.47±3.51^b^	10.24±1.48^a^	13.26±2.08^a^
Whole blood	HbA1c (%)	15.7±4.74^a^	8.62±2.44^b^	35.8±6.27^c^	27.6±5.86^d^
Liver	TG (mg/g)	8.96±1.77^a^	8.78±1.52^a^	21.4±3.39^b^	53.9±10.92^c^
	Glycogen (mg/g)	5.63±1.77^a^	5.63±1.77^a^	10.8±2.44^b^	8.53±1.29^c^
Muscle	TG (mg/g)	3.52±0.41^a^	3.07±0.34^a^	14.3±2.07^b^	14.6±1.42^b^
	Glycogen (mg/g)	1.21±0.30^a^	1.19±0.29^a^	1.00±0.16^a^	1.01±0.17^a^

^a,b,c,d^ Those means not sharing a common letter in the same row are significantly different between groups (n = 8) (P <0.05).

*The values are expressed as the means±SD.

### Effect of CGA on the content of BW, body fat, FPG, HbA1c and insulin

At the end of the experiment, compared with the *db/db*-CON group, the VA percentage in *db/db*-CGA was significantly reduced by 10% (P <0.05), glycogen in the liver was 21% lower (P <0.05), TG in the liver was significantly higher (P <0.05), and levels of FPG and HbA1c were significantly decreased by 28.8% and 22.9%, respectively (P <0.05). However, plasma TG, TC, Lee's index, and the insulin level were not significantly different between the two groups (P> 0.05). In db/m mice, Lee's index and the HbA1c level in the CGA group were significantly decreased by 42% and 45.2%, respectively (P <0.05), whereas the levels of insulin and FFA were significantly increased in the CGA group (P <0.05). The percentages of VA, FPG, TG and glycogen in both muscle and liver were not significantly different between the two groups (P> 0.05) (Table 1).

### Effect of CGA on OGTT

**Fig 1:**
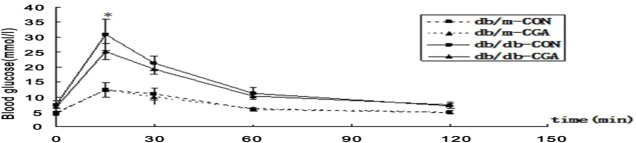
OGTT Test on *db/db* and db/m Mice after 8 Weeks of Intervention with CGA. An oral glucose tolerance test was performed on *db/db* and db/m (n = 8 each) mice. 2.5 g/kg glucose was loaded at 0 minutes. Blood samples were collected at 0, 15, 30, 60 and 120 min for glucose measurements. The values are expressed as the mean±SD. *P <0.05.

At the end of the experiment, the OGTT results showed that the 15-min peak blood glucose level in the CGA group was decreased by 18.2% (P <0.05) compared with the control group of *db/db* mice. There were no differences at 30 min, 60 min or 120 min between the two groups (P> 0.05). The glucose AUC in the CGA group showed a non-significant decrease of 10.5% (P> 0.05). The level of plasma glucose was not significantly different in the db/m mice at all time-points (Fig. 1).

### Effect of CGA on the level of adiponectin and visfatin in VA in *db/db* and db/m mice

At the end of the experiment, compared with the control group, the adiponectin level in VA was 50.2% higher (P <0.05), and the visfatin level was 32.7% (P <0.05) lower in the CGA group in *db/db* mice. However, both the levels of adiponectin and visfatin were not different in the db/m mice between the two groups (P >0.05) (Table 2).

**Table 2:** Visfatin and adiponectin levels of visceral adipose and aldose reductase activity of the kidney in *db/db* and db/m mice groups.*

****Items****		****db/m-CON****	****db/m-CGA****	*****db/db*****-CON****	***db/db*-CGA**
Visceral adipose	Visfatin (μg/mg)	31.1±9.0^a^	26.4±6.1^a^	100±16.1^b^	67.3±11.8^c^
	Adiponectin (μg/mg)	520±45.4^a^	547±54.4^a^	242±49.6^b^	363±19.8^c^
Kidney	AR activity (μmol/mg/min)	0.185±0.03^a^	0.174±0.03^a^	0.687±0.15^b^	0.484±0.09^c^

^a,b,c^ Those means not sharing a common letter in the same row were significantly different between groups (n = 8) (p<0.05).

*The values are expressed as the means±SD.

### Effect of CGA on the activity of AR and protein expression of TGFβ-1 in the kidney of *db/db* and db/m mice

**Fig 2:**
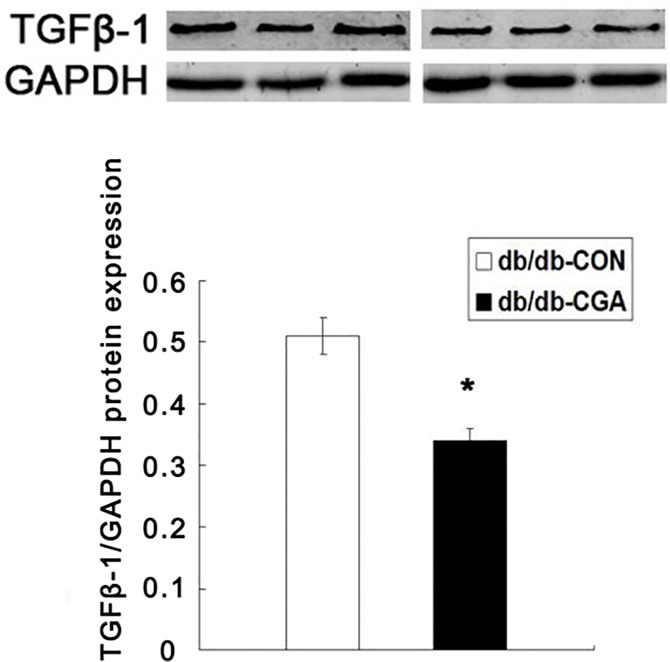
CGA Lowered the Protein Expression of TGFβ-1 in *db/db* Mice. *P <0.05 compared with the *db/db*-CON group (n = 8).

At the end of the experiment, the AR activity of the kidney in *db/db* mice was significantly higher than in the db/m group (P <0.05). Compared with the control group, the activity of AR and the protein expression of TGF-β1 were 29.5% and 33.3% lower, respectively, in the CGA group of *db/db* mice (P <0.05). The AR activity of the kidney was not different in db/m mice from the two groups (Table 2) (Fig. 2).

### Effect of CGA on protein expression of ADPNR-2, pAMPK, and G-6-P in the liver of *db/db* mice

**Fig 3:**
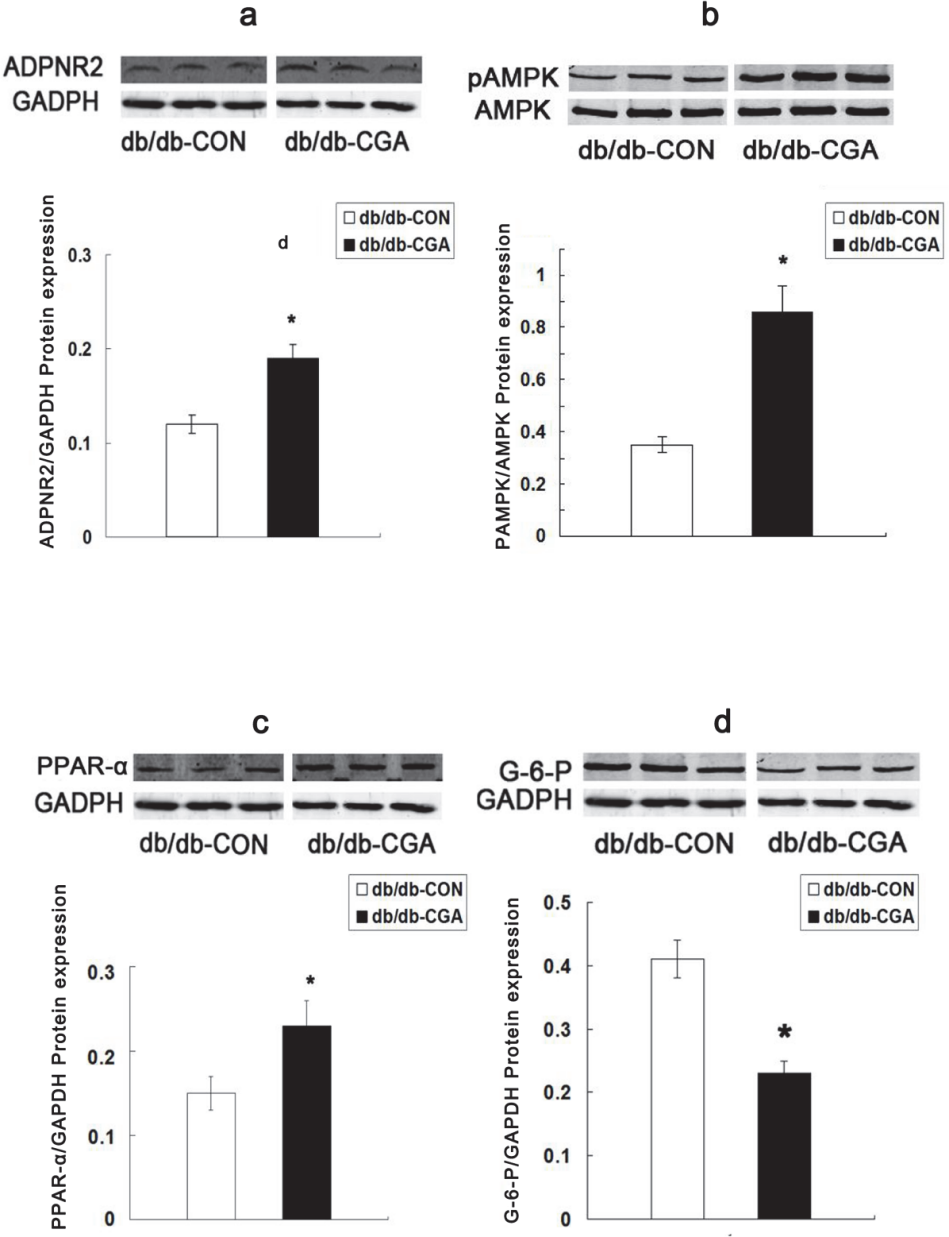
Effects of CGA on the Protein Expression of ADPNR-2 (a), pAMPK (b), PPAR-α (c), and G-6-P (d) in the Liver of *db/db* Mice. *P <0.05 compared with the *db/db*-CON group.

After 12 weeks of intervention, compared with the control group, the liver protein expression of ADPNR-2 (Fig. 3A) and AMPK phosphorylation (Fig. 3A) were 58.3% and 143% higher in the CGA treatment group (P <0.05). In contrast, the liver protein expression of G-6-P was 43.9% lower in the CGA treatment group (P <0.05) (Fig. 3D).

### Effect of CGA on mRNA and protein expression of PPAR-α in the livers of *db/db* and db/m mice

**Fig 4:**
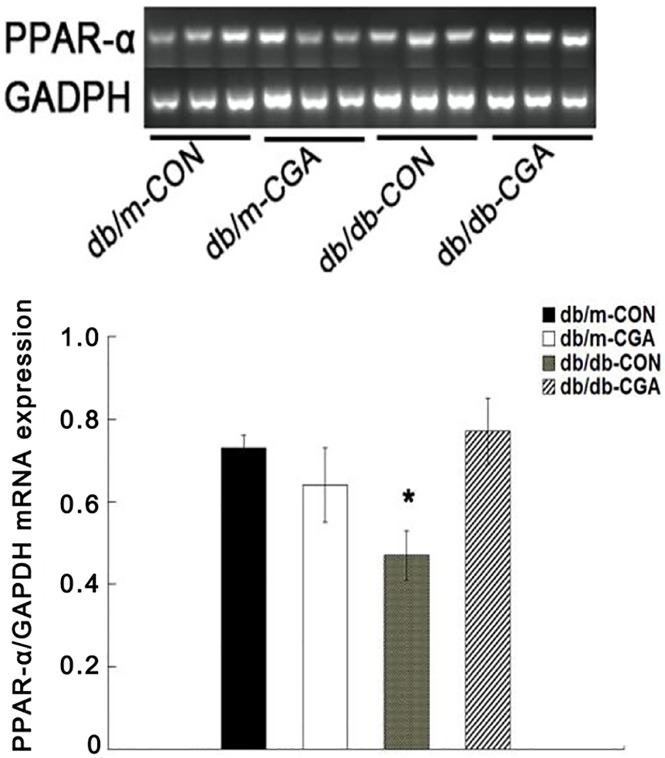
CGA Effects on PPAR-α mRNA Expression in the Liver of *db/db* and db/m Mice. *P <0.05 compared with the *db/db*-CON group, n = 8.

After 12 weeks of intervention, compared with the control group, CGA administration significantly up-regulated the mRNA (Fig. 4) and protein (Fig. 3C) expression of PPAR-α by 64.3% and 53.3%, respectively, in the liver of *db/db* mice (P <0.05). The expression of mRNA in the liver was not different from that of the db/m mice (P >0.05) (Fig. 4).

### Effect of CGA on protein expression of ADPNR-1, pAMPK, PPAR-α, and GLUT-4 in the skeletal muscle of *db/db* mice

**Fig 5:**
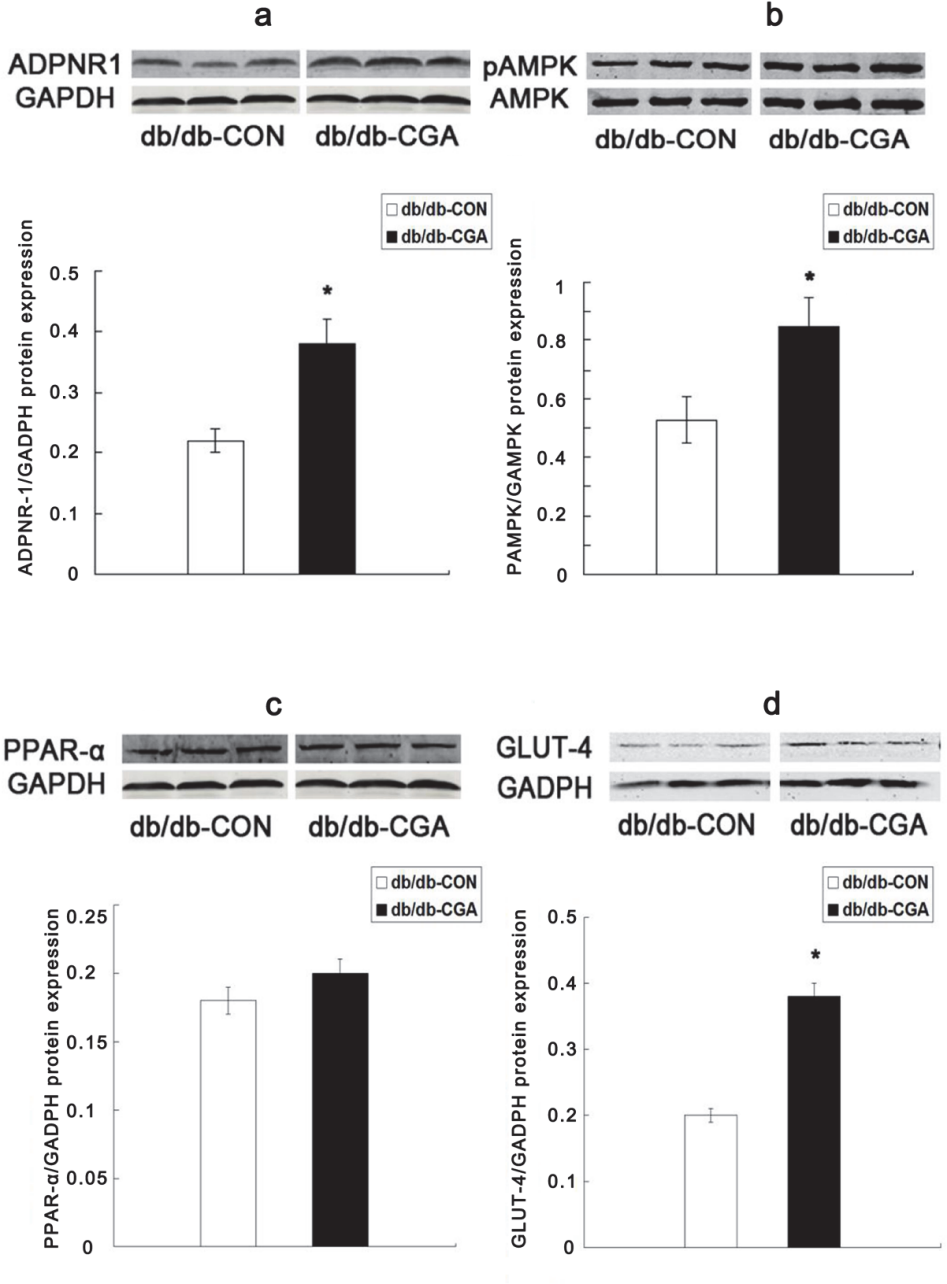
Effect of CGA on the Protein Expression of ADPNR-1 (a), pAMPK (b), PPAR-α (c), and GLUT-4 (d) in the Muscle of *db/db* Mice. *P <0.05 compared with the *db/db*-CON group.

After 12 weeks of intervention, compared with the control group, the skeletal muscle protein expression of ADPNR-1 (Fig. 5A), AMPK phosphorylation (Fig. 5B), and GLUT-4 (Fig. 5D) were significantly higher by 72.7%, 60.4% and 89.5%, respectively, in the CGA treatment group of *db/db* mice (P <0.05). The protein expression of PPAR-α was not significantly different in the *db/db* mice (P >0.05) (Fig. 5C).

## Discussion

In this study, we used *db/db* mice as a type 2 diabetic mouse model to observe the impact of CGA on glucose and lipid metabolism disorders and diabetic nephropathy. The results showed that CGA significantly reduced FPG and HbA1c in *db/db* mice and also improved fibrosis in diabetic nephropathy (DN). Its mechanism may be related to the regulation of adiponectin receptors and related signaling molecules.

*Db/db* mice have a homozygous spontaneous mutation of the leptin receptor and are a diabetic model that shows type 2 diabetic characteristics such as obesity at 3–4 weeks after birth, an uncontrolled rise in blood glucose and insulin 4–8 weeks after birth, typical diabetic nephropathy and serious loss of pancreatic β cells. Plasma insulin declined 15–17 weeks after birth, and the process of type 2 diabetes in this model is similar to that in human beings [23, 24]. Although different genetic mutations in db/db mice can induce variation in phenotype, but this article can reflect a part of the whole picture. In this study, compared with db/m mice, the weight, Lee’s index and VA percentage as well as the TG in the plasma, liver and skeletal muscle were significantly higher in *db/db* mice, conforming to the model of spontaneous obese diabetes. Between the two groups of *db/db* mice and db/m mice, food-intake was not significantly different, indicating that CGA did not affect the food-intake in either *db/db* or db/m mice.

The aim of OGTT test was to evaluate the function of islet β-cell. Previous studies found CGA can significantly low OGTT glucose AUC and postprandial glucose [15, 25]. After 8 weeks of intervention in our study, CGA significantly reduced the 15-min peak blood glucose in an OGTT (P <0.05) but did not significantly reduce the blood glucose at other times, nor the glucose AUC, indicating that CGA might lower transient blood sugar, but could not improve the insulin level which was secreted by islet β-cell in db/db mice. At the end of 12 weeks, CGA had no significant effect on the level of plasma insulin, which is consistent with our previous study in male *db/db* mice [16]. However, the FPG, HbA1c and visceral adipose content were all reduced in the *db/db*-CGA group. Therefore, we speculated CGA may act its function through inslulin sensitization pathway.

At the same time, we also found CGA increased adiponectin and decreased visfatin in visceral fat (P <0.05). Recent studies in L6 myotubes by Ong et al. found an additive effect of CGA on insulin-mediated glucose transport, suggesting that CGA may act through a significant pathway that is different from insulin signaling [20]. Above all, CGA may exert its anti-diabetic function through adiponectin receptor-mediated signaling pathway. As we all know the increase in visceral adipose tissue is closely related to diabetes, lipid metabolism disorders and hypertension but negatively correlated with adiponectin secretion [26]. As a key cytokine in visceral adipose, even at extremely low physiological concentrations in the liver, adiponectin is a very effective insulin sensitizer, and elevated levels in the plasma are positively correlated with insulin sensitivity [27]. Low serum adiponectin has been linked to central obesity, insulin resistance, type 2 diabetes, and metabolic syndrome [28]. Both male and female workers with or without type 2 diabetes in Japan who regularly drink coffee rich in CGA have higher plasma adiponectin, and they show a dose-dependent association between coffee consumption and adiponectin level [29–31]. Unfortunately, our blood samples were too small to measure plasma adiponectin. We had to detect adiponectin in adipose tissue, which is a maior source of adipokines. In the research of Yuichiro Kawano, both plasma adiponectin and mRNA expression in white adipose tissue were significantly elevated in male Zucker diabetic fatty rats who were treated by a popular procure for treating obesity [32]. Further research is needed to clarify the relevance of these findings.

In the present study, we showed for the first time an effect of CGA on adiponectin receptors in the liver and muscle of the *db/db* diabetic animal model. CGA increased ADPNR-2 in the liver by 58.3% and ADPNR-1 in muscle by 72.7%. Previous studies indicated that ADPNR-1 and ADPNR-2 serve as major ADPNRs *in vivo* [21]. Adiponectin primarily combines with the c-terminus first, which is distributed on ADPNR-1 in muscle and ADPNR-2 in the liver, and then bonds with a signal adapter protein through the membrane via the N-terminal, thereby activating downstream signal transduction pathways [33]. The expression of ADPNR-1 and ADPNR-2 are decreased both in obese mice and humans, and decreased ADPNRs in obesity can reduce adiponectin sensitivity, which ultimately leads to diminished insulin sensitivity, whereas up-regulating ADPNRs can ameliorate the symptoms of diabetes. At the same time, ADPNR function relies on adiponectin [21, 34]. In our study, CGA up-regulated the ADPNRs in the liver and muscle, and thus CGA may improve glucose and lipid disorders in diabetic animals through adiponectin receptors.

There are two pathways for ADPNRs to exert their function. One is to accelerate the β-oxidation of fatty acids in the liver and muscle via PPAR-α, which is a member of the nuclear receptor family of ligand-activated transcription factors. Liver and muscle are the main target organs, and their key role is to enhance enzyme activity during the process of fatty acid β-oxidation [35]. Regardless of rodent type, a high-fat diet with CGA can up-regulate PPAR-α in the liver and subsequently enhance the enzyme activities involved in fatty acid oxidation [13, 17]. PPAR-α is one of the key molecules downstream of the ADPNRs that can also up-regulate the expression of ADPNRs [21]. In our previous work [16], both the mRNA and the protein levels of PPAR-α were significantly upregulated in *db/db*-CGA male mice, in full agreement with this study.

Another function is to regulate glucose and lipid metabolism in liver and muscle through AMPK phosphorylation [21, 34]. AMPK is a type of protein kinase that plays a critical role in cellular energy status and systemic energy balance [36, 37] and can be activated through a variety of means [20]. Active AMPK can act on multiple signaling molecules downstream. In the liver, it can enhance lipid metabolism by increasing the activities of acetyl coenzyme A carboxylase (ACC) and PPAR-α and also inhibit the activity of G-6-P [20, 37]. In skeletal muscle, the activation of AMPK causes GLUT4 translocation, thereby increasing glucose intake [38, 39]. Prabhakar et al. showed that CGA stimulates glucose transport in myotubes by increasing the expression of GLTU4 [40]. Three years later, Ong et al. investigated the role of CGA in *db/db* mice and L6 skeletal muscle, demonstrating that CGA stimulates glucose transport by activating AMPK [20]. Further, chronic administration of CGA inhibits hepatic G-6-P expression and activity [41]. In our study, CGA significantly up-regulated phospho-AMPK in liver and skeletal muscle, and down-regulated G-6-P in liver, while up-regulating GLUT4. Finally, FPG, HbA1c and visceral adipose content were decreased by CGA. In summary, these results indicate that CGA potentially ameliorates glucose and lipid disorders in diabetic mice through ADPNR mediated signaling pathways, and it may play a role in maintaining whole-body homeostasis. More research is needed to support the use of CGA to target ADPNR-mediated signaling in the prevention and treatment of type 2 diabetes.

Another important finding in our study was that CGA may improve renal fibrosis in late diabetic *db/db* mice. DN has long been recognized to cause severe morbidity and mortality. An important aspect in improving the quality of life in diabetic patients is to prevent and delay the emergence and worsening of DN. *Db/db* mice exhibit significant renal pathobiology at 10–20 weeks old [24]. After 12 weeks of intervention, our results showed that AR in *db/db* mice was three times higher than in db/m but that CGA significantly decreased the activity of AR and down-regulated the protein expression of TGFβ-1 (P <0.05) in the *db/db* mice. The activation of AR, a key rate-limiting enzyme in the polyol pathway, and increased expression of TGFβ-1 in the kidney are the most important factors in the development of DN. Hyperglycemia causes the increase in AR activity, resulting in the accumulation of intracellular sorbitol, ultimately causing the destruction of structure and function in the target tissue. The inhibitor of AR activity can significantly improve DN [42]. Recent research *in vitro* showed that CGA significantly inhibited AR activity [43, 44]. Hyperglycemia in diabetes stimulates the increase in TGFβ-1 expression and, in turn, induces kidney multi-cellular hypertrophy through autocrine and paracrine pathways. Hyperglycemia was also a key indicator in assessing the degree of renal fibrosis in DN [45, 46]. The inhibition of AR activity and TGFβ-1 expression has an important role in improving diabetic nephropathy. The reason may be that CGA improved insulin sensitivity through an ADPNR mediated signaling pathway. The decreased level of FPG, HbA1c and postprandial blood sugar may have significant functions in ameliorating DN.

In addition, during the 12 consecutive week intervention, we did not find that CGA had any adverse effects in db/m mice, but we did find that CGA significantly reduced the visceral fat content and increased the plasma insulin levels in db/m mice. In this study, the hepatic TG was elevated in *db/db*-CGA mice. In the study by Li et al., CGA decreased the levels of TG in plasma and liver in high-fat diet-fed golden hamsters but had no effect on LPL activity, showing that excessive exogenous fat flows into the liver to be metabolized. However, lipopexia in liver was critically high in 15-17-week-old *db/db* mice after birth. CGA, as a natural phyto-compound, cannot successfully clear haptic TG. Only one article has been published about overdose intake of CGA (2 g/d), in which it caused elevated blood homocysteine [40]. Under conditions of acute peritoneal injection or chronic feeding in rats, no side effects of overtaking CGA have been observed [41, 47]. Whether CGA can cause an increase in hepatotoxicity and enhance steatosis needs more research. All these results provide a safety and efficacy evaluation for CGA in the prevention and treatment of type 2 diabetes.

The limiting factors in this study were that the sample size was not enough to detect the plasma adiponectin level and the plasma insulin at the end of 8 weeks intervention as well as the terminal stage level of OGTT. More research is needed to confirm this mechanism.

In summary, we have demonstrated that CGA decreased the fasting plasma glucose, glycosylated hemoglobin and visceral fat contents and improved renal fibrosis in *db/db* diabetic mice. The mechanism may occur through an adiponectin receptor-mediated signaling pathway as follows. CGA elevates the adiponectin level in visceral fat and the adiponectin receptors in liver and muscle in *db/db* mice. Furthermore, CGA reduces the activity of G-6-P in the liver to inhibit gluconeogenesis and increase glucose transport in skeletal muscle through the phosphorylation of AMPK and improves disordered lipid metabolism through PPAR-α. The most important finding in our study is that the long-term administration of CGA may improve glucose and lipid metabolism disorders in late diabetic mice. Moreover, it also may prevent diabetic complications. Our findings combined with the evidence from other studies strongly suggest that CGA, as a natural phytochemical, may contribute, at least in part, to the beneficial effect of coffee on the blood glucose levels of patients with type 2 diabetes.
